# Impact of the Tumor Microenvironment on Tumor Heterogeneity and Consequences for Cancer Cell Plasticity and Stemness

**DOI:** 10.3390/cancers12123716

**Published:** 2020-12-11

**Authors:** Ralf Hass, Juliane von der Ohe, Hendrik Ungefroren

**Affiliations:** 1 Biochemistry and Tumor Biology Lab, Department of Obstetrics and Gynecology, Hannover Medical School, 30625 Hannover, Germany; Ohe.Juliane.von.der@mh-hannover.de; 2 First Department of Medicine, University Hospital Schleswig-Holstein, Campus Lübeck, 23538 Lübeck, Germany; hendrik.ungefroren@uksh.de; 3 Department of General Surgery, Visceral, Thoracic, Transplantation and Pediatric Surgery, University Hospital Schleswig-Holstein, Campus Kiel, 24105 Kiel, Germany

**Keywords:** cancer cell fusion, mesenchymal stroma/stem-like cells, post-hybrid selection process, retrodifferentiation, activated and silenced cancer stem cell niche, tumor plasticity, epithelial-mesenchymal transition, cancer stem cells

## Abstract

**Simple Summary:**

The cancer cells in solid tumors are embedded in a complex connective tissue matrix composed of various other cell types, i.e., mesenchymal stroma/stem-like cells (MSCs) and tumor-associated macrophages (TAMs). This tumor microenvironment (TME) is considered the major cause of tumor heterogeneity, which in turn accounts for treatment failure in current cancer therapies. Physical and chemical signals from the TME as well as factors secreted by MSCs and TAMs can induce epigenetic alterations in the cancer cells that alter their phenotypic plasticity, eventually resulting in the generation of cancer stem cells (CSCs). Phenotype switching of CSCs involves processes such as epithelial-mesenchymal transition, transdifferentiation, retrodifferentiation, or spontaneous cell fusion of cancer cells with stromal cells, particularly MSCs. Principally, phenotype plasticity of cancer (stem) cells may be targeted pharmacologically to reduce tumor heterogeneity and hence resistance to therapy.

**Abstract:**

Tumor heterogeneity is considered the major cause of treatment failure in current cancer therapies. This feature of solid tumors is not only the result of clonal outgrowth of cells with genetic mutations, but also of epigenetic alterations induced by physical and chemical signals from the tumor microenvironment (TME). Besides fibroblasts, endothelial and immune cells, mesenchymal stroma/stem-like cells (MSCs) and tumor-associated macrophages (TAMs) intimately crosstalk with cancer cells and can exhibit both anti- and pro-tumorigenic effects. MSCs can alter cancer cellular phenotypes to increase cancer cell plasticity, eventually resulting in the generation of cancer stem cells (CSCs). The shift between different phenotypic states (phenotype switching) of CSCs is controlled via both genetic programs, such as epithelial-mesenchymal transdifferentiation or retrodifferentiation, and epigenetic alterations triggered by signals from the TME, like hypoxia, spatial heterogeneity or stromal cell-derived chemokines. Finally, we highlight the role of spontaneous cancer cell fusion with various types of stromal cells. i.e., MSCs in shaping CSC plasticity. A better understanding of cell plasticity and phenotype shifting in CSCs is a prerequisite for exploiting this phenomenon to reduce tumor heterogeneity, thereby improving the chance for therapy success.

## 1. Introduction

**Figure 1:**
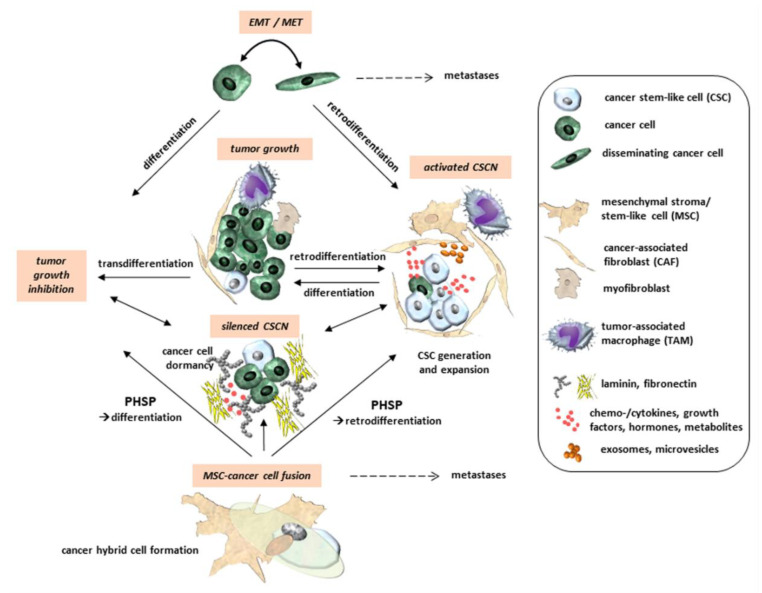
Tumor plasticity comprises several genetic and epigenetic programs including epithelial-mesenchymal transition (EMT)/ mesenchymal-epithelial transition (MET) or cancer cell fusion. These contribute to the enrichment of cancer cell progenitors or cancer stem cells (CSC) populations within an activated CSCN (cancer stem cell niche) for CSC expansion or a silenced CSCN for maintenance of dormant CSCs. The multiple underlying programs such as differentiation, trans- or retrodifferentiation, metastases formation, or PHSP (post-hybrid selection process) may take place simultaneously in distinct compartments of the tumor tissue (adapted from [18,26]).

**Figure 2:**
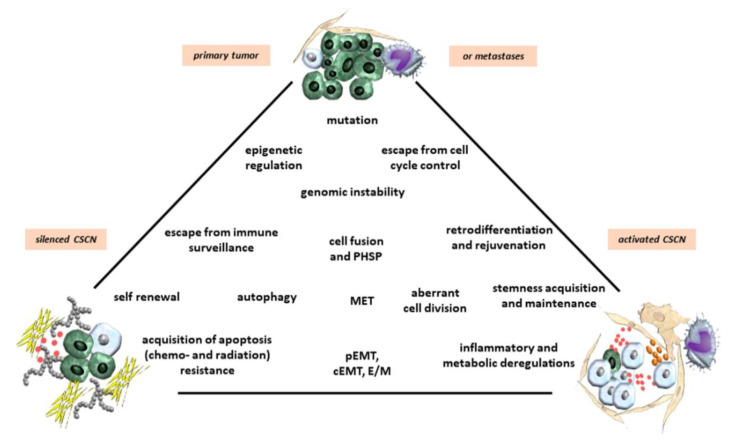
Various pathways and mechanisms can contribute to cancer cell plasticity during shuttling between (I) an activated cancer stem cell niche (CSCN) with cancer cell progenitor and CSC expansion, (II) a silenced CSCN displaying cancer cell dormancy, and (III) the dynamic alterations in the primary tumor tissue and metastases. Symbols are described in Figure 1.

Solid tumors is composed of cancer cells interacting with a variety of non-tumorigenic cells such as immune cells (e.g., T cells, natural killer cells, macrophages), endothelial cells, adipocytes, mesenchymal stroma/stem-like cells (MSCs), and fibroblasts which are embedded in a distinct matrix of structural proteins constituting the extracellular matrix (ECM). These cellular components together with the ECM form the tumor microenvironment (TME), which promotes the development and expansion of cancer progenitor cells, tumor-initiating cells (TICs), and cancer stem cells (CSCs) [1,2]. The TME of solid tumors is subject to dynamic turnover of its structural and functional components, and this process partially accounts for the phenomenon of tumor heterogeneity. CSCs and TICs were identified and characterized in several human primary and metastatic neoplasms such as ovarian [3], prostate [4], breast [5], lung [6], and pancreatic cancer [7], melanomas [8], acute myeloid leukemia [9], glioblastoma [10,11,12], and other brain tumors [13]. TICs, CSCs, and their progeny are suggested to reside in specialized compartments termed cancer stem cell niche (CSCN) and according to their functionalities discrimination between activated and silenced CSCNs is hypothesized. Protection and progression of progenitor or CSC populations requires an activated CSCN as a distinct transient location in certain tissues (primary tumor tissue or metastatic tissue). Alternatively, CSCN-like structures in an inactivated more silenced state may also harbor quiescent CSCs or diverse CSC-derived progeny following cell cycle exit into a transiently growth-arrested G_0′_-like phase by entering dormancy (Figure 1 and Figure 2). Accordingly, CSCs may be distinguished by dormancy-competent, cancer-repopulating, dormancy-incompetent, and disseminated populations [14] whereby tumor dormancy can be induced by various processes including metastasis, radiation/chemotherapy, and cancer cell fusion among others [15]. This hibernation-like state of CSCs enables survival by escape from immune surveillance and maintaining treatment resistance until CSCN conversion into an activated state and reentry of CSCs into the proliferative state, eventually followed by tumor recurrence [16]. CSCNs can be reversibly established by mediators such as prostaglandin E2 signaling [17] and compartmentalized by interacting cell types involving MSCs and cancer-associated fibroblasts (CAFs) [18,19,20].

During mutual interactions within the TME the cellular partners can change their tasks. In particular, interactions of macrophages or MSCs with cancer cells play an important role in modulating tumor functions, thereby increasing tumor heterogeneity [21]. For instance, macrophages, particularly M2-type macrophages, are converted to TAMs (tumor-associated macrophages) [22,23] and MSCs can differentiate into CAFs (cancer-associated fibroblasts) [24,25] displaying mostly tumor-supportive properties (Figure 1). MSCs can also indirectly and directly interact with cancer cells by modulating their functions and contributing to cancer cell plasticity [19,20].

## 2. Cancer Cell Plasticity and CSCs

Cell plasticity is defined as the ability of a cell to reprogram and change its phenotypic identity, a phenomenon also known as lineage plasticity. Cell plasticity occurs in several fundamental biological processes, such as embryonic development, wound healing, tissue regeneration, or neoplastic transformation. In cancer, the reactivation of these mechanisms enables tumor cells to acquire a CSC-like phenotype with enhanced ability to escape apoptosis in hostile environments, thereby contributing to cancer initiation, progression, metastases, and therapy resistance [27,28]. Cancer are phenotypically plastic and may stochastically, or in response to environmental cues, adopt CSC and non-CSC states in a dynamic and reversible fashion, eventually giving rise to different subsets of CSCs (Figure 1). The different phenotypes of CSCs and TICs enlarge plasticity and are determined by maintenance of stemness, self-renewal capability, escape from immune surveillance, and resistance to apoptosis induced by chemotherapeutic drugs. Cancer cell plasticity is also induced by intrinsic/cell-autonomous genetic and/or epigenetic alterations and by extrinsic factors such as dynamic restructuring within the TME [29,30,31,32,33].

Besides displaying remarkable genetic/epigenetic, metabolic, and phenotypic heterogeneity, cancer cells maintain plasticity by transition along a spectrum of cellular phenotypes intermittent between the extreme epithelial or mesenchymal states in a process regulated by the TME. Deregulated/aberrant cell plasticity could be considered another hallmark of a cancer.

Together with their self-renewal capacity, immune escape, and resistance to chemotherapeutic interventions CSCs contribute to tumor maintenance and development. Thereby, CSCs can differentiate along various pathways and initiate new tumors [34]. CSCs are characterized by the expression of distinct markers. In renal cell carcinoma, expression of the stem cell protein CD133 also known as prominin-1 and the hyaluronan receptor CD44 are associated with CSCs [35]. Breast CSCs represent a combination of the GPI-anchored sialoglycoprotein CD24, aldehyde dehydrogenase-1 (ALDH-1), and CD44 in a CD24^low^/CD44^high^/ALDH^high^ constellation [5]. CSCs in colorectal tumors are characterized by a set of proteins such as CD44, CD133, CD24, EpCAM, LGR5, and ALDH [36]. Detection of CSCs by the expression of corresponding markers in other tumors include pancreatic carcinoma (epithelial-specific antigen (ESA), CD24, CD44) [7], medulloblastomas and gliomas (CD133) [13], epithelial ovarian cancers (CD117 (c-kit), CD44) [3], malignant melanoma (ATP-binding cassette sub-family B member 5 (ABCB5)) [8], prostate cancer (CD133, α2β1 integrin, CD44) [4], lung cancer (CD133) [6], among others. These and more different CSC marker profiles suggest the appearance of tumor type- and tumor tissue-specific CSC populations.

CSCs may also differ from TICs as the cell of tumor origin in their phenotypic and molecular characteristics [37,38,39,40,41]. The cell of tumor origin acquires the first cancer-initiating mutational hit, i.e., TICs [38]. According to the hierarchical model this tumor-originating cell could be a normal native/lineage stem cell [41], or a committed progenitor or differentiated cell as hypothesized by the stochastic model [18,37]. Examples of cells of origin include the Lgr5+ stem cell type of the intestinal crypt for colorectal cancers [42] or acinar cells that convert through a process termed acinar-to-ductal metaplasia (ADM), to pancreatic ductal adenocarcinoma (PDAC) [43]. ADM is a prominent example for the process of transdifferentiation, the direct conversion of a one differentiated cell into a functionally altered type of differentiated cell without passing through an intermittent retrodifferentiation and progenitor/stem-like state.

Alternatively, committed progenitors or differentiated cells which undergo a retrodifferentiation program reacquire stem cell features by losing previously functional identities resulting in a CSC phenotype [27,28,39,44,45]. Retrodifferentiation is characterized by a reversion of maturated properties and expression patterns of a differentiated phenotype to a precursor cell or stem-like cell [46,47,48]. In addition, retrodifferentiation to a stem-like state includes retrograde senescence, providing longevity or rejuvenation at the cellular level [49]. CSCs which are generated by retrodifferentiation from differentiated cells regain capacity for self-renewal and may thus be able to maintain tumorigenicity [50,51]. When cells exhibit plasticity, they converge on signaling processes that induce cellular retrodifferentiation (Figure 1 and Figure 2). Activation of these programs, in turn, enables the initiation and progression of carcinogenesis and underlies resistance to therapy [52]. Interactions of cancer cells with MSCs or CAFs can induce a retrodifferentiation program to form CSCs [53,54]. This enables new differentiation pathways, whereby CSCs maturate along an altered lineage [51]. Whereas MSCs contribute to the establishment of CSCs to maintain and promote CSC growth [18], intrinsic processes such as EMT or pharmacological interference with (trans)differentiation of TICs or CSCs may induce maturation and growth reduction followed by reduced tumorigenicity [55].

Accumulating evidence suggests that certain cancer cells can adopt a CSC state associated with hybrid/partial EMT, a higher transdifferentiation potential, and increased resistance to chemo- or radiotherapy [56,57,58]. At the molecular level, resistance is acquired by several different mechanisms, including upregulation/activation of multidrug efflux pumps, enhanced DNA repair, or maintenance of a slow cycling, or a quiescent state [59,60]. The process of tumor re-initiation or recurrence by stem-like cells presumably involves differentiation of quiescent or dormant CSCs into rapidly proliferating tumor cells. Slow cycling or quiescent stem-like cells in the tumor contribute to resistance against conventional chemo- and radiotherapies because these treatments are usually directed towards rapidly dividing cells, substantiating the hypothesis of activated and silenced CSCNs (Figure 1 and Figure 2).

CSCs also exhibit a remarkable ability to reprogram their cellular metabolism in response to signals they receive from the TME [30,61]. Although the metabolic phenotypes of CSCs have been insufficiently characterized so far, the stem-like features resulting from altered metabolic pathways is another emerging hallmark of cancer, that contributes to CSC plasticity [62,63]. Various cell-intrinsic and cell-extrinsic factors may modulate this “metabostemness” and CSCs can transit from one metabolic state to another in response to various conditions in the TME, like pH, hypoxia, or nutrient supply [61,64]. A better understanding of the association between metabolic phenotype and plasticity of CSCs is required in order to exploit the underlying mechanisms for effectively targeting these cells.

## 3. Epigenetic Reprogramming of CSCs

Cancer cell phenotype shifting and CSC generation are largely controlled by epigenetic mechanisms that render the chromatin restrictive or permissive for specific transcriptional programs involved in cell differentiation or reprogramming [28,34,58,65] (Figure 2). Signature patterns of “active” chromatin marks active promoters, transcribed regions, and candidate enhancers, whereas other modifications reveal distinct modes of chromatin repression, such as those mediated by the Polycomb repressor complex 2 (PRC2). In CSCs, a bivalent chromatin state may enhance plasticity and phenotypic switching [28,34,58,65]. For instance, the promoter hyper-methylation-induced silencing of HOXC8 (a homeobox gene), in non-tumorigenic mammary epithelial cells has been found to be associated with an increase in the number of CSCs, heightened self-renewal and a transformed phenotype [66]. A histone modifier, enhancer of zeste homolog 2 (EZH2), the catalytic subunit of PRC2, induces transcriptional repression of target genes via trimethylation of lysine-27 in histone H3 (H3K27me3) [67]. In CSCs of various malignant tumors EZH2 is expressed at markedly elevated levels and exhibits a crucial function in CSC maintenance and progression [68]. In breast cancer (BC) cells, EZH2 promotes expansion of TICs and mammosphere formation through activation of RAF1/β-catenin signaling [69,70], while in glioblastoma loss of H3K27me3 can lead to aberrant Wnt signaling activation, which is necessary for maintenance of CSCs [71]. In contrast, missense mutations in the genes encoding histone H3.3 and H3.1. in pediatric glioblastomas have lower overall amounts of H3K27me3 due to inhibition of the enzymatic activity of PRC2 through interaction with the EZH2 subunit, an epigenetic dysregulation that may promote gliomagenesis [72].

Epigenetic alterations can drive oncogenic programs even in the absence of mutations in classical oncogenes or tumor suppressor genes [73]. In addition, certain chromatin structures can inhibit differentiation and prevent appropriate induction of tumor suppressor programs. By contrast, permissive or “plastic” states, i.e., via enhancer landscape reprogramming during tumorigenesis [74] may allow random oncogene activation or non-physiologic cell fate transitions and therapy resistance in cancer cells [65]. Both TME-driven and epigenetic reprogramming promote such dynamic mechanisms, favoring cancer cell plasticity and tumor heterogeneity [39,75]. Current evidence points to a complex interplay between the genes, epigenetic changes and the TME in cell reprogramming, cancer cell plasticity, and tumor heterogeneity [39,76]. These observations suggest that epigenetic changes triggered by interactions with the TME modulate cancer cell phenotypes and properties, and shape tumor architecture [77]. Moreover, CSCs are capable of exploiting the reversible nature of epigenetic modifications to adjust their plastic state. Similar to the diverse EMT phenotypes, this reversibility may be harnessed for therapeutic targeting.

## 4. Regulatory Effects of the TME and Its Cellular Components on CSCs

### 4.1. The Effect of Inflammation and Hypoxia on CSC Plasticity

Chronic inflammation represents a hallmark of many cancers and inflammatory signals contribute to tumor initiation, progression and cell plasticity [34]. The tissue healing process is accompanied by remodeling of the local tissue environment, which changes the spectrum and production of various growth factors, enzymes and other signaling mediators secreted by specific niche cells and the surrounding stromal cells, such as immune cells and MSCs. Evidence for an association between inflammation and cell plasticity comes from a variety of cancer types [45,78,79,80]. A rare population of long-lived, quiescent pancreatic cells labeled by doublecortin-like kinase-1 (Dclk1) is required in vivo for pancreatic regeneration following injury and chronic inflammation. Expression of mutant Kras in Dclk1+ cells converts these cells into potent TICs upon induction of experimental pancreatitis [80]. Another study with aged mice deficient in the homeobox gene, *Nkx3.1*, revealed that loss of function of *Nkx3.1* accelerates inflammation-driven prostate cancer initiation potentially via aberrant cellular plasticity and impairment of cellular differentiation [81]. A hypoxic microenvironment is known to regulate various aspects of malignant progression including cellular plasticity. For instance, hypoxia was found to promote self-renewal in non-stem cells in glioblastoma by upregulating OCT4, NANOG, and c-MYC [82]. Moreover, a hypoxic TME in vivo favors the accumulation of cells with CSC-like features. The authors concluded that this was due to clonal evolution or selection, since the differential phenotypes of the tumor cells from both the hypoxic and non-hypoxic TMEs were fairly stable even when subsequently cultured in vitro under normoxic culture conditions [31]. These studies underscore the importance of orchestrating components within the TME in generating intra-tumoral heterogeneity and CSC plasticity, knowledge that is crucial for the rational design of novel and more effective therapeutic strategies. However, it still remains unclear whether CSC heterogeneity is a consequence of selection pressure exerted by the TME or whether plasticity represents an inherent feature of the cancer cells that enables them to adapt to varying signals from the TME [39,83]. Recent evidence from a study on glioblastoma suggests that the expression of CSC-associated membrane markers is based on intrinsic plasticity of tumor cells rather than on clonal entity defined by distinct functional properties or transcriptomic profiles. These authors showed that phenotypic heterogeneity arose from non-hierarchical, reversible state transitions that were triggered by the TME. They went on to conclude that tumorigenic potential is not coupled to CSC multipotency but to intrinsic plasticity and, hence, that therapies directed against CSC-associated membrane epitopes may fail [83].

The physical and chemical composition of the TME with parameters such as pH, oxygen content, ion concentrations, nutrient availability, and mechanic rigidity of the ECM also plays a significant role in establishing a CSCN and regulating CSC behavior [84,85,86]. The dynamic restructuring of the tumor stroma affects CSCN establishment and maintenance. Activated or silenced CSCN structures can be dismantled at certain tumor sites and newly build up at other more favorable places within the tumor tissue, suggesting a variety of simultaneous opportunities for CSCNs to provide a CSC compartment (Figure 1 and Figure 2). This reversible construction and degradation of a CSCN depends on the form and the stability of the local TME compartment and associated tissue composition, but also applies to primary tumors and metastases. For example, CSCNs of tumor metastases in the hypoxic bone marrow are more protected and stabilized in rigid and spongy bone cavities as compared to CSCNs in metabolically-exposed tissues such as primary organ-associated tumor tissues or soft-tissue lymph node metastases. Consequently, bone marrow-associated compartments may represent a preferable location for silenced CSCNs and CSC dormancy which is supported by long-term residing cancer cells in bone metastases.

A fundamental feature of the TME is spatial heterogeneity [87,88], which is able to impact cellular phenotypes. For instance, glioblastoma cells residing in hypoxic regions of a tumor overexpress EGFR (epidermal growth factor receptor), while vascular regions were enriched in platelet-derived growth factor receptor α [89]. Similarly, a hypoxic TME induced by anti-angiogenic agents can increase breast CSCs [90]. Thus, spatial heterogeneity of TME can give rise to generation of cancer subpopulations at different locations within an individual tumor. Spatial heterogeneity in primary tumors has also been reported with respect to cells with EMT [91,92,93]. Cells at the invasive front of the primary tumor as well as metastases were deficient in the expression of membrane-bound E-cadherin but express high levels of nuclear β-catenin, suggestive of an EMT (Figure 1). In contrast, the more centrally located cells in the primary tumor and in metastases were rich in membranous E-cadherin and cytoplasmic β-catenin, probably indicative of mesenchymal-epithelial transition (MET) [94]. Likewise, mesenchymal breast CSCs were found at the invasive edge of the tumor, while the more epithelial or hybrid epithelial/mesenchymal (E/M) CSCs were localized in its central regions [93]. In oral squamous carcinoma, budding cells exhibited a particular gene expression signature when compared to cells from the central parts of tumors that comprise factors involved in EMT and activated TGF-β signaling. ZEB1 was upregulated concomitantly with the decreased expression of MET-associated transcription factors (TFs), e.g., OVOL1, Krüppel-like factors and Grainyhead-like factors. Moreover, microRNA-200 family members were found to be downregulated in budding tumor cells [95]. Using a mechanism-based dynamical model it was shown that the more mesenchymal CSCs lie at the invasive front, while the hybrid E/M CSCs reside in the central regions of the tumor. The mathematical simulations also revealed that the diffusion of TGF-β (a strong EMT-inducer) along with Notch signaling-mediated control of EMT can provide an explanation for the differential localization of CSC subpopulations with varying EMT phenotypes in the tumor [32]. Spatial heterogeneity of tumors may be used as a prognostic marker for treatment response across different cancer types [88]. Moreover, TGF-β-activated SMAD TFs contribute to a fibrogenic EMT program involving Ras-responsive element binding protein 1, a transcriptional effector of activated HRAS and KRAS. This complex induces SNAIL expression which can promote subsequent intratumoral fibrosis and tumor growth [96].

### 4.2. The Effects of Stromal Cells of the TME on Cancer Cell EMT and Plasticity

#### 4.2.1. CAFs and TAMs in EMT and Tumors

Accumulating evidence suggests that cancer cell/stromal cell intercellular communications strongly impact stem-like behavior and phenotypic plasticity of cancer cells [97,98,99]. As mentioned above, CAFs are a major component of the TME and play a fundamental role in various aspects of tumor progression [100]. These cells were found to regulate the plasticity of TICs in hepatocellular carcinomas through c-Met/FRA1/HEY1 signaling [101], in PDAC through p125^FAK^ signaling [102], and in lung cancer by insulin-like growth factor receptor signaling [103].

A series of studies on various cancers highlight the contribution of CCL chemokine expression to the activation of EMT programs [104]. CCL2-mediated monocyte/macrophage trafficking was also observed in the inducible Kras^G12D^p53-null PDAC mouse model [33]. Subsequently, TGF-β secreted by recruited TAMs induces tumor cells to adopt a mesenchymal phenotype. This enabled them to survive extinction of oncogenic Kras, indicating a significant role of the CCL2-TGF-β/EMT signaling pathway in the resistance to Kras-targeted therapy [33].

In response to chemotherapy, macrophages can secrete Oncostatin-M (OSM), an IL-6 family cytokine, which—via activation of STAT3/SMAD3 signaling [105]—induces the retrodifferentiation of triple-negative breast cancer cells into aggressive stem cells [106]. OSM is also secreted by cancer-associated adipocytes, which are likewise able to promote stemness [107]. Li and colleagues studied how differently polarized M1 or M2 macrophages communicate with epithelial-mesenchymal plasticity of cancer cells, and vice versa, how cancer cells in an epithelial or mesenchymal state can influence the polarization of macrophages [108]. Using in silico co-culture models it was found that the interactions between cancer cells and macrophages can give rise to multiple stable steady-states, with each steady-state being stable against external perturbations. More recently, in a transgenic mouse model of ovarian carcinoma, it was demonstrated that the chemoresistance-promoting functions of TAMs require expression of Zeb1 by TAMs with the release of CCL2 by the cancer cells [109]. As discussed above, expression of Zeb1 by cancer cells endows them with a more aggressive phenotype, including enhanced invasive capacities, therapeutic resistance, and stemness, resulting in poor clinical outcomes in a variety of human cancer types [110]. Clinical trials are currently designed with cancer cell-expressed ZEB1 as a potential molecular target. However, the above data suggest that effective inhibition of tumor growth and improved response to chemotherapy would also require targeting ZEB1 in TAMs [109]. Similar contributions by TAMs to the resistance to cytostatic drugs via EMT induction have been observed in other cancer types, including pancreatic and colorectal cancers [111,112]. Hence, strategies targeting TAM function, infiltration, or activation can be exploited for therapeutic purposes.

#### 4.2.2. MSC Origin and Role in Tumors

MSCs represent a heterogeneous mixture of subpopulations also termed multipotent mesenchymal stromal cells or medicinal signaling cells [113,114,115]. While the underlying basis of MSC heterogeneity remains unclear, previous work suggested that this mixed population may be explained by mutual interdependency of different stromal cell clones. These subclones adapt their availability by clonal convergence or expansion to maintain growth potential of the entire population and to provide the various MSC properties within progressively changing environments such as tumor stroma [20]. Different organs also exhibit a tissue-specific environment for MSCs which adds to their variable characteristics. Primary origins of MSCs are localized in perivascular regions of various adult tissues [116,117]. In addition, MSCs can also be isolated from neonatal tissues such as placenta or umbilical cord in large quantities. These MSC populations originating from birth-associated tissues exhibit superior in vitro growth potential and regenerative capacity [118].

Together with other subpopulations displaying stem-like characteristics, MSCs are functionally involved in tissue repair and regenerative activities [115,119,120,121]. Primary MSC cultures enable in vitro maintenance for a limited time as compared to constitutively proliferating MSC-like cells, representing a cell source with permanently reproducible properties [122,123]. Following recruitment to cancer cell-induced lesions, the regenerative potential of MSCs operates in promoting tissue repair during invasive tumor growth. MSCs thereby, develop both tumor-inhibiting [124] and tumor-promoting properties [19,125,126] during crosstalk with cancer cells and other neighboring tumor-associated populations within the TME [127]. MSCs interact with a variety of immune cells and exhibit immune-modulatory functions. They suppress the cytotoxic capacity of NK cells [128], inhibit T cell activation, and contribute to a conversion of inflammation-associated M1 to repair-oriented M2(a–d) macrophages by altering immune cell functions and favoring immune suppression [129,130,131,132].

Activation of MSC’s paracrine capabilities produces a variety of chemokines, growth factors, and metabolites which are secreted into the TME, e.g., by induction of endothelial cells to support tumor vasculogenesis [133]. Conversely, the phenotypes of cancer cells are altered under the influence of MSCs, e.g., via release of TGF-β to promote tumor growth by acquisition of novel properties and to mediate differentiation of endothelial cells for enhanced tumor angiogenesis [134]. Moreover, delivery of TGF-β can also induce an epithelial-mesenchymal transition (EMT) in cancer cells, eventually promoting the generation of new CSCs by retrodifferentiation (Figure 1). EMT cells can also be reversed by the mesenchymal-epithelial transition (MET) program, i.e., during outgrowth of macrometastases at distant sites (see below).

Besides these more indirect, paracrine communication pathways, MSCs directly interact with cancer cells, for example via gap junctional intercellular communication [125]. Connexins as the molecular monomers can form a homo- or heterohexameric hemichannel in the plasma membrane which can assemble to a corresponding hemichannel of an adjacent cell to build a gap junction. These structures play an essential role in the transcellular exchange of ions, metabolites, and second messengers and also affect cytoskeletal signaling. A variety of different connexins are distributed among cancer cells modulating proper communication and contributing to heterogeneity by tumor inhibition or tumor progression. Since tumor-type- and stage-specific compositions of connexins can be determined, these membrane proteins possess tumor prognostic value and are a useful molecular target for therapeutic interventions [135]. Further examples for direct interactions between MSCs and cancer cells include the formation of F-actin-rich tunneling nanotubes or trogocytosis to exchange molecules, small organelles or cell membrane patches [19,53]. Moreover, notch receptor signaling [125] is involved in maintaining self-renewal and amplification of CSCs. Previous work has demonstrated that the range of intracellular Notch1 signaling in MSC-derived dermal fibroblasts governs the capability of these cells to regulate melanoma aggressiveness, stemness, and phenotypic plasticity [136].

Following close cellular interactions spontaneous cell fusion between MSCs and cancer cells with the generation of new hybrid cancer cell populations represents a rare event in tumors which can also display CSC characteristics [137,138,139,140,141] (Figure 1). Several distinct molecular mechanisms can contribute to cell fusion in a cell type-specific manner with tight membrane approaches as a prerequisite to enable a fusogenic environment. Fused hybrid cells undergo a post-hybrid selection process (PHSP) to enable chromosomal rearrangements for successful cell cycle progression and a renewed accurate cell metabolism [142] (see below).

### 4.3. Cancer Cell Fusion and CSC Plasticity

Besides mutual communication via soluble and physical means, close cellular interactions eventually result in spontaneous cell fusion, for example between macrophages/TAMs and cancer cells [143,144,145], or between MSCs/CAFs and cancer cells [137,141,146,147]. During fusion, the newly formed hybrid cancer cells express different DNA profiles and acquire diverse functional characteristics from the parental cells. For example, the macrophage-specific factors DAP12 [148] and CD163 [149], both of which are not detectable in breast cancer cells, are expressed in fused hybrid breast cancer cells [150].

**Figure 3:**
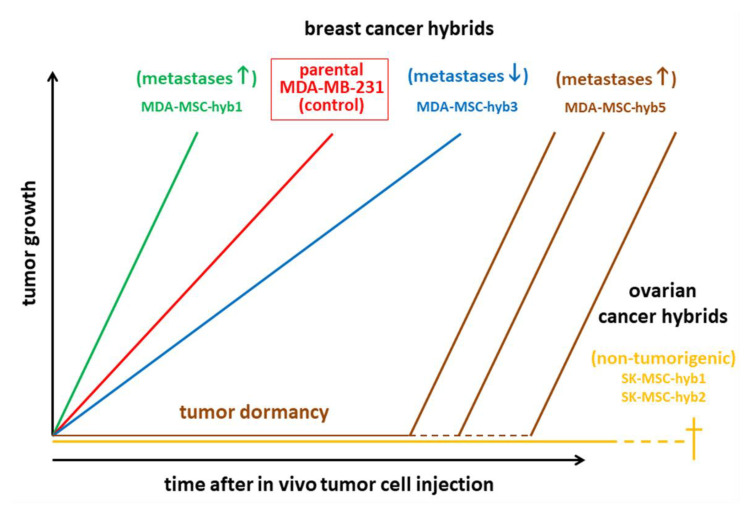
Tumor development of different human breast cancer hybrid cells demonstrates enhanced (green) and reduced (blue) growth properties as compared to the parental breast cancer cells (red) before fusion with mesenchymal stroma/stem-like cells (MSCs) and subsequent PHSP. A third form of tumor growth is characterized by an initial phase of dormancy. The two presented ovarian cancer hybrid populations displayed no detectable tumor development and the animals eventually died of old age.

Cancer cell fusion with the generation of new hybrid cancer cell populations increases tumor plasticity by the generation of subpopulations with CSC-like traits [21,137,138,139,141,151]. Hybrid cancer cells, however, can either enhance tumorigenicity and metastatic capacity [152] or reduce neoplastic behavior [138,153] (Figure 1, Figure 2 and Figure 3). The acquisition of novel functionalities and increased tumor plasticity is determined by a PHSP leading to hybrid cancer cells with different tumorigenic and metastatic behavior [142]. This selection program is required since fusion-derived cancer hybrid cells generate aneuploid or polyploid giant cells displaying uncoordinated nuclear and DNA communication [154]. Further processes underlying the generation of aneuploidy include endoreplication, endomitosis, aging, or engulfment by entosis [155], or cannibalism [156]. The accompanying chromosomal instabilities require appropriate reduction to a (meta)stabilized level that is regulated during a PHSP. This multistep program of PHSP displays a clonal convergence of the initial hybrid population by elimination, silencing, or stabilizing surviving hybrids [142]. Thereafter, PHSP-generated hybrid cancer cells enhance tumor plasticity by newly acquired functionalities including CSC-like properties, which affect tumor growth and metastatic spreading (Figure 3). Indeed, cancer cell fusion contributes to the development of CSC subtypes [157]. The resulting tumor heterogeneity complicates therapeutic regimen, suggesting unfavorable patient outcome (Figure 1, Figure 2 and Figure 3).

Cancer cell fusion with MSCs can also reduce tumorigenic properties as demonstrated by MDA-MSC-hyb3 and –hyb4 breast cancer cells [138] and SK-MSC-hyb1 and –hyb2 ovarian cancer hybrids [153] (Figure 3). Moreover, the aggressive tumor-promoting hybrids MDA-MSC-hyb1 and –hyb2 demonstrate increased vulnerability to various chemotherapeutic compounds such as taxol, cisplatin, methotrexate, epirubicin, and foretinib [152], suggesting that fusion of cancer cells with MSCs causes distinct therapy-oriented effects, that are not observed during cancer cell fusion with macrophages.

These findings underscore the need for a better mechanistic understanding of the fusion process and subsequent PHSP to predict and potentially regulate the outcome and functionality of hybrid cells. Cancer cell fusion in different cancer cell types, however, involves different molecular mechanisms. For instance, MSC fusion with neoplastic MCF-10A mammary epithelial cells can be promoted by tumor necrosis factor α (TNFα) and blocked by inhibition of TNF receptor [137]. Alternatively, occurrence and progression of a rhabdomyoblastoma by cancer cell fusion was suppressed by inhibition of the interleukin-4 receptor [158]. This mechanistic heterogeneity among different tumor types complicates common therapeutic strategies and successful interventions.

## 5. Exploiting Cancer Cell Plasticity for Improving Therapeutic Success

In the past few years, cell plasticity has emerged as a mode of escape from targeted and non-targeted therapies in various cancers. However, our understanding of this phenomenon has also expanded, raising hope that vulnerabilities associated with tumor cell plasticity may be harnessed for the development of novel and innovative therapeutic concepts. Alone or in combination with existing anticancer treatments, these could lead to more complete and longer-lasting clinical responses. In this last section, we comment on translational aspects citing a selection of preclinical and clinical studies mainly in malignant melanoma providing some proof that targeting tumor cell plasticity is a viable therapeutic option. Further in-depth discussions on this issue are provided by two recent reviews [26,29].

Experimental animal models: Melanoma plasticity is linked to phenotype switching, where the TME induces switches between invasive vs. proliferative states. In a zebrafish model, melanoma cells following extravasation activate genes of melanocyte differentiation and become pigmented. After metastatic dissemination, the TME provides signals in the form of EDN3 to promote phenotype switching, which can induce a state that is both proliferative and differentiated and associated with decreased animal survival [159]. The smoothened inhibitor, vismodegib/GDC0449, has been shown to induce tumor shrinkage of basal cell carcinoma (BCC) by promoting tumor cell differentiation. However, a small subpopulation of tumor cells survives and accounts for tumor relapse following treatment interruption, a situation seen also in humans. In both mouse and human BCC, this persisting, slow-cycling subpopulation is characterized by expression of the stem cell marker, LGR5, and active Wnt signaling. Employing GEMMs of BCC, Sánchez-Danés and coworkers showed that the combination of vismodegib treatment with Lgr5 lineage ablation, or an inhibitor of Wnt signaling, led to tumor eradication, demonstrating that the synergy between Wnt and Smoothened inhibitors is a clinically feasible strategy to overcome tumor recurrence in BCC [160].

Patient-derived biological material: Human melanoma cells can display profound transcriptional variability at the single-cell level, which involves high-level transcription in a very small percentage of cells of a number of genes encoding resistance markers. This set of marker genes and the related gene expression signatures reveal the prognostic relevance for defining transcriptional phenotypes/states that in turn predict, which cells will ultimately resist drug treatment [161]. Another study highlights the potential of therapies directed towards minimal residual disease (MRD). In malignant cells isolated from BRAF mutant melanoma PDXs exposed to RAF/MEK inhibitors, varying combinations of distinct drug-tolerant transcriptional states were identified, which co-existed within MRDs from PDXs and biopsies of patients under therapy. Among these, the authors were able to identify a novel neural crest stem cell (NCSC) transcriptional program and the nuclear receptor RXRG as key drivers of resistance by showing that inhibiting RXRG prevented the accumulation of NCSCs in MRD and delayed the generation of resistant cells [162].

Continuous BRAF inhibition of *BRAF* mutant melanomas is known to induce a series of phenotypic changes in the cancer cells that result in therapy resistance and escape from immune control before genetic fixation of the acquired resistant state. This is due to activation of certain signaling networks shortly after BRAF inhibition, but before the appearance of drug-resistant phenotypes. Drug targeting those networks, in combination with BRAF inhibition, halted the adaptive transition and led to prolonged growth inhibition in multiple patient-derived cell lines [163]. Also in melanoma, AXL^high^ cells are resistant, while AXL^low^ cells are sensitive to MAPK pathway inhibitors, rationalizing a differential therapeutic approach. To achieve this goal, Boshuizen and colleagues developed an antibody-drug conjugate, AXL-107-MMAE that as a single agent displayed potent in vivo anti-tumor activity in PDXs derived from melanoma, lung, pancreatic and cervical tumors. Moreover, in combination with MAPK inhibitors, AXL-107-MMAE eliminated distinct populations in heterogeneous melanoma cell pools and inhibited tumor growth. These findings provide proof-of-concept that rationalized combinatorial targeting of distinct populations in heterogeneous tumors can improve therapeutic efficiency [164].

Clinical trials: In melanoma, MITF and its upstream activator, PAX3, are drivers of an early non-mutational and reversible drug-tolerant state. Nelfinavir has been identified as a potent suppressor of PAX3 and MITF expression and sensitizer of BRAF and NRAS mutant melanoma cells to MAPK pathway inhibitors. Moreover, nelfinavir is effective in BRAF and NRAS mutant melanoma cells isolated from patients progressed on MAPK inhibitor therapy and in BRAF/NRAS/PTEN mutant tumors [165]. As discussed above, PRC2 has been shown to play a major role in transcriptional silencing in part by methylation of H3K27 and deregulation of its function correlates with poor prognosis in certain cancers. Vaswani et al. have identified CPI-1205, a highly potent and selective inhibitor of EZH2, the active subunit of PRC2, that displayed robust antitumor effects in a xenograft models and is currently evaluated in phase 1 and 1b/2 clinical trials, alone and in combination [166], (https://www.constellationpharma.com/constellation-pharmaceuticals-announces-first-patient-dosed-phase-1b-2-prostar-combination-study-cpi-1205-advanced-form-prostate-cancer/). Two other selective EZH2 inhibitors, Tazemetostat and GSK2816126, are currently subject to phase-1 clinical testing. While the Tazemetostat trial is being conducted in patients with relapsed or refractory B-cell non-Hodgkin lymphoma and advanced solid tumors [167], GSK2816126 is being performed with relapsed/refractory diffuse large B cell lymphoma, transformed follicular lymphoma, other Non-Hodgkin’s lymphomas, multiple myeloma, and castrate-resistant prostate cancer (https://www.clinicaltrials.gov/ct2/show/NCT02082977). The above referenced studies impressively show that therapeutic manipulation intended to increase or decrease tumor heterogeneity and cancer cell plasticity, either alone or in combination with conventional therapies, holds great promise in overcoming therapy resistance and improving patient outcomes.

## 6. Conclusions

Enhanced recruitment of MSCs or TAMs to the tumor tissue increases the chance of mutual interactions with the cancer cells via soluble mediators, physical interactions or even cell fusion, eventually resulting in cancer cell EMT, retro-/transdifferentiation, and the generation of various CSC phenotypes besides their contribution to establish CSCNs. These cellular programs or the signaling peculiarities that distinguish hybrid cancer populations from their parental counterparts and the intermediate states during a PHSP represent potential therapeutic targets to direct cancer cell development along a more differentiated and less tumorigenic path [129,153,168,169]. Transfer of chromosomes from non-tumor-associated MSCs during fusion with cancer cells may provide more tumor-reducing or anti-tumor properties in contrast to cancer cell fusion with macrophages. Deciphering the underlying mechanisms of cancer cell fusion, CSC development, maintenance in activated or silenced (dormant) CSCNs, and repopulating/differentiating capabilities is crucial for a better understanding of tumor plasticity. This will pave the ground for novel therapeutic strategies that alone or in combination with conventional therapies will hopefully overcome therapy resistance and improve patient outcomes.

## Abbreviations

ADM	acinar-to-ductal metaplasia
CAFs	cancer-associated fibroblasts
CSCs	cancer stem cells
CSCN	cancer stem cell niche
CTCs	circulating tumor cells
ECM	extracellular matrix
E/M	epithelial/mesenchymal
EZH2	enhancer of zeste homolog 2
EMT	epithelial-mesenchymal transition
MET	mesenchymal-epithelial transition
MSC	mesenchymal stroma/stem-like cells
PDAC	pancreatic ductal adenocarcinoma
PHSP	post-hybrid selection process
PRC2	polycomb repressor complex 2
TAMs	tumor-associated macrophages
TFs	transcription factors
TICs	tumor-initiating cells
TME	tumor microenvironment
TNF	tumor necrosis factor
Zeb1/2	zinc finger E-box-binding 1/2
